# Mechanistic Protective Effect of Cilostazol in Cisplatin-Induced Testicular Damage via Regulation of Oxidative Stress and TNF-α/NF-κB/Caspase-3 Pathways

**DOI:** 10.3390/ijms241612651

**Published:** 2023-08-10

**Authors:** Eman M. Othman, Heba A. Habib, Mahmoud E. Zahran, Amr Amin, Gehan H. Heeba

**Affiliations:** 1 Department of Biochemistry, Faculty of Pharmacy, Minia University, Minia 61519, Egypt; eman@toxi.uni-wuerzburg.de; 2 Department of Bioinformatics, Biocenter, University of Wuerzburg, Am Hubland, 97074 Wuerzburg, Germany; 3 Department of Pharmacology and Toxicology, Faculty of Pharmacy, Minia University, Minia 61519, Egypt; dr.hebaadel@yahoo.com; 4 Minia Oncology Center, Minia 61511, Egypt; mehab5669@gmail.com; 5 Biology Department, College of Science, UAE University, Al-Ain 15551, United Arab Emirates

**Keywords:** cisplatin, cilostazol, tadalafil, pentoxifylline, testicular damage

## Abstract

Despite being a potent anticancer drug, cisplatin has limited applicability due to its adverse effects, such as testicular damage. Consequently, reducing its toxicity becomes necessary. In this study, a selective phosphodiesterase-3 inhibitor, cilostazol, which is used to treat intermittent claudication, was examined for its ability to abrogate cisplatin-induced testicular toxicity. Its ameliorative effect was compared to that of two phosphodiesterase inhibitors, tadalafil and pentoxifylline. The study also focused on the possible mechanisms involved in the proposed protective effect. Cisplatin-treated rats showed a significant decrease in sperm number and motility, serum testosterone, and testicular glutathione levels, as well as a significant elevation in malondialdehyde, total nitrite levels, and the protein expression of tumor necrosis factor-alpha, nuclear factor-kappa β, and caspase-3. These outcomes were confirmed by marked testicular architecture deterioration. Contrary to this, cilostazol, in a dose-dependent manner, showed potential protection against testicular toxicity, reversed the disrupted testicular function, and improved histological alterations through rebalancing of oxidative stress, inflammation, and apoptosis. In addition, cilostazol exerted a more pronounced protective effect in comparison to tadalafil and pentoxifylline. In conclusion, cilostazol ameliorates cisplatin-induced testicular impairment through alteration of oxidative stress, inflammation, and apoptotic pathways, offering a promising treatment for cisplatin-induced testicular damage.

## 1. Introduction

Cisplatin (CIS) is a potent platinum chemotherapeutic compound that is widely used in the treatment of various solid tumors. However, renal toxicity, hepatotoxicity, and gonadal toxicity restrict its use. After CIS treatment, approximately all patients suffer from testicular atrophy and azoospermia, which disrupts the social lives of their partners. Therefore, the prevention of CIS-related reproductive toxicity is a major challenge [1,2,3].

Despite the lack of knowledge regarding the exact pathophysiological mechanisms of CIS-induced gonadal injury, several earlier studies have revealed that direct DNA damage and oxidative stress in testicular tissue may be associated with its toxicity. Overproduction of reactive oxygen species (ROS) triggers inflammation-inducing mediators, such as tumor necrosis factor-alpha (TNF-α) and reactive nitrogen species (RNS), which consequently causes necrosis and apoptosis of testicular cells [4,5]. Therefore, the alleviation of these underlying mechanisms is a rational strategy to hinder CIS-induced reproductive toxicity.

Cyclic adenosine monophosphate (cAMP) is a central intracellular second messenger involved in multiple intracellular signaling pathways [6]. It regulates cell differentiation, growth, and gene transcription in testicular cells. Moreover, cAMP is the intracellular messenger for the steroidogenesis action of luteinizing hormone [7]. Regulation of cAMP is performed by a superfamily of enzymes called phosphodiesterases (PDEs). However, inhibition of PDEs increases cAMP levels, which enhances cell function [8]. In recent decades, different phosphodiesterase inhibitors have offered powerful protective effects against a variety of testicular diseases [9,10,11,12]. The potential chemoprotective effect of tadalafil (TDF) [13,14], a selective long-acting PDE-5 inhibitor, and pentoxifylline (PTX) [15], a nonspecific PDE-4 inhibitor, has been demonstrated against CIS-induced reproductive toxicity in males.

Meanwhile, cilostazol (Cilo), a selective PDE-3 inhibitor, abrogates platelet aggregation, while also acting as a vasodilator and an antithrombotic [16]. Cilo is a commonly prescribed medication for intermittent claudication caused by peripheral arterial disease [17]. According to recent studies, Cilo possesses pleiotropic effects that include antioxidant, anti-apoptotic, and anti-inflammatory properties [11,18,19,20,21]. Moreover, it has been shown to protect against different models of testicular injury, including testicular ischemia reperfusion [21] and diabetic testicular damage [11]. In these studies, Cilo significantly improved testicular inflammation and oxidative stress states.

To the best of our knowledge, the impact of Cilo on CIS-induced male reproductive toxicity has not been clarified. The purpose of this study was to determine whether Cilo (in a dose-dependent manner) protects against testicular damage induced by CIS and unravel the underlying mechanisms. In addition, Cilo’s proposed therapeutic potential against CIS-induced androgenic toxicity was compared here to other PDE inhibitors.

## 2. Results

### 2.1. Effect on Relative Testicular Weight, Testosterone Concentration, and Epididymal Sperm Parameters

As listed in Table 1, the relative weights of the testes in the CIS group were significantly (*p* < 0.05) lower than those of the control group. As compared to the CIS group, the relative testicular weight was significantly (*p* < 0.05) higher in CIS-intoxicated rats treated with TDF, PTX, and different doses of Cilo. In addition, the CIS group exhibited a significant decrease in the serum testosterone level, sperm count, and motility, as well as a significant increase in the percentage of sperm with abnormal morphology in comparison to control animals.

However, the impairment in testosterone concentration and the measured sperm parameters were significantly abolished with pre-conditioning with TDF, PTX, and Cilo (5, 10, and 20 mg/kg). Interestingly, the middle dose of Cilo (10 mg) caused a significant improvement in serum testosterone levels, the motility of epididymal sperm, and the percentage of abnormal sperm morphology when compared to the CIS + Cilo 5 mg group. Also, with the increasing dose of Cilo up to 20 mg/kg, a significant increase in the testosterone concentration and sperm indices were observed as compared to the CIS + Cilo 10 mg group.

In comparing the effect of 20 mg/kg of Cilo with TDF and PTX in the CIS-treated groups, 20 mg/kg of Cilo caused a significant elevation in the testosterone concentration, sperm count, and motility, along with a significant decrease in the percentage of sperm with abnormal morphology.

### 2.2. Histopathological Findings of Testicular Tissues

**Figure 1:**
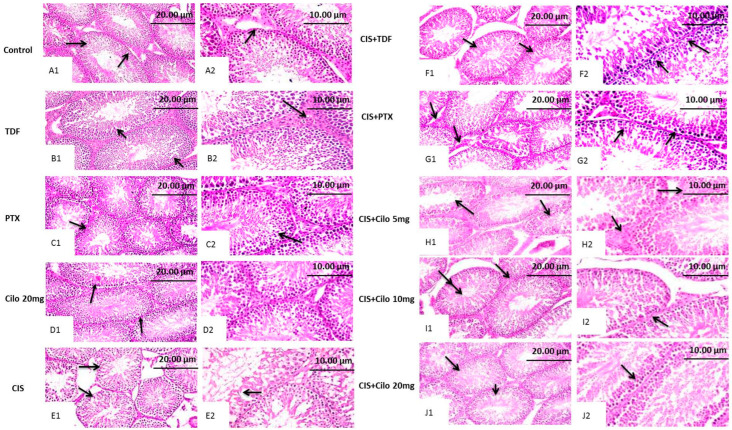
Effect of cilostazol (5, 10, or 20 mg/kg), tadalafil (5 mg/kg), and pentoxifylline (75 mg/kg) on testicular tissues stained with hematoxylin and eosin in CIS-treated rats. Photomicrograph of testis from control, TDF, PTX, and Cilo 20 mg groups showing active spermatogenesis in normal-size seminiferous tubules with thin basement membranes (arrow, (**A1**,**B1**,**C1**,**D1**), ×200) and intact interstitial cells in between (arrow, (**A2**,**B2**,**C2**,**D2**) ×400). Testicular tissues of CIS-treated rats presenting disorganized spermatogenic cells within distorted, shrunken seminiferous tubules (arrow, (**E1**), ×200) and reduced germinal cell layers, along with hyperactivity of Sertoli cells (arrow, (**E2**), ×400). Photomicrograph of a testis obtained from the CIS + TDF group showing partial reduction in the seminiferous tubule size, with depletion of germinal cells (arrow, (**F1**), ×200) and nuclear pyknosis of spermatogenic cells (arrow, (**F2**), ×400). Testicular tissue from the CIS + PTX group represents an eosinophilic edematous area in between seminiferous tubules, with a reduction in interstitial cell numbers (arrow, (**G1**), ×200) and germinal cell degeneration, with pyknotic nuclei (arrow, (**G2**), ×400). Testis tissue micrographs of the CIS + Cilo 5 mg group exhibit mild disorganization of spermatogenic cells within empty tubules from spermatids (arrow, (**H1**), ×200) and edema in between seminiferous tubules with attenuated interstitial cell numbers (arrow, (**H2**), ×400). Testis tissue of the CIS + Cilo 10 mg group represents a reduction in the size of seminiferous tubules, with a widened interstitial space (arrow, (**I1**), ×200), and pyknosis of germinal cells and depletion of interstitial cells (arrow, (**I2**), ×400). The CIS + Cilo 20 mg group showed improvement in histological findings, with regular arrangement of germinal cells within the seminiferous tubules (arrow, (**J1**), ×200) and a normal tubular lumen containing spermatids (arrow, (**J2**), ×400). CIS: Cisplatin; TDF: Tadalafil; PTX: Pentoxifylline; Cilo: Cilostazol.

Representative images of H&E-stained testicular sections showed active spermatogenesis and normal-size seminiferous tubules in the testicular parenchyma of the control, TDF, PTX, and Cilo 20 mg groups. Each tubule was surrounded by an outer thin layer of connective tissue and lined with spermatogenic cells, which consisted of primary and secondary spermatocytes, spermatogonia, and spermatids. These tubules were separated by interstitial cells in clusters characterized by large and ovoid nuclei (Figure 1A–D).

In contrast, the seminiferous tubules of the CIS-treated group revealed progressive histological abnormalities (Figure 1E). Sections obtained from the testes of the CIS group revealed severely disorganized spermatogenic cells, reduced germinal cell layers, and interstitial cells of distorted, shrunken seminiferous tubules. Histological alterations, such as pyknotic nuclei and a vacuolated cytoplasm, were observed in spermatogenic cells. Moreover, spermatogenic cell apoptosis appeared as deeply eosinophilic bodies with hyperactivity of Sertoli cells.

Contrary to this, the histological damage in testicular components markedly improved with all used drugs (Figure 1F–J). Moreover, the CIS + Cilo 20 mg group exhibited great protection of seminiferous tubules compared to groups treated with lower doses of Cilo or the other two drugs. Most of the histopathological lesions seen in the testicular tissues of CIS-intoxicated rats disappeared in the group of rats protected by a high dose of Cilo. The scoring of testicular tissue injury in the 10 groups is illustrated in Table 2.

### 2.3. Effect on Testicular Malondialdehyde, Total Nitrite Content, and Reduced Glutathione

**Figure 2:**
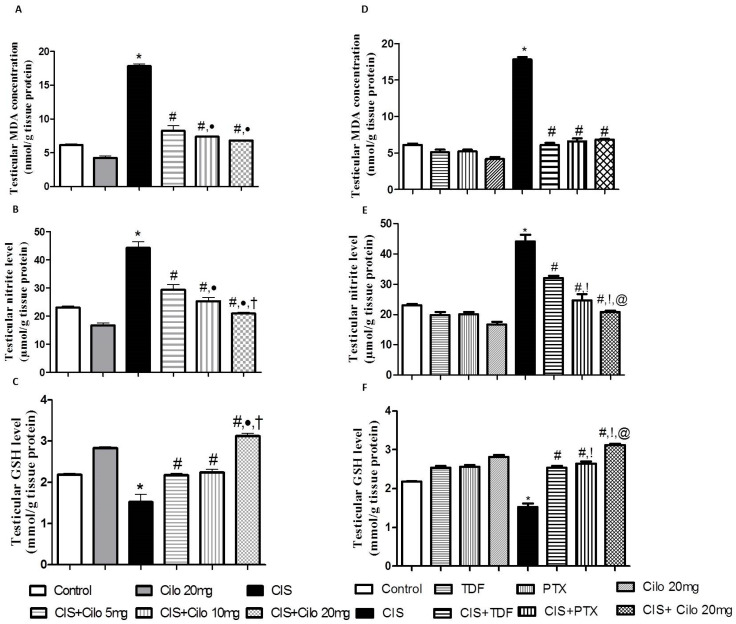
Effect of cilostazol (5, 10, and 20 mg/kg) on oxidative stress indicators in a CIS-induced testicular dysfunction rat model, accompanied by comparison between the levels of oxidative stress indicators in rat testes of groups treated with tadalafil (5 mg/kg), pentoxifylline (75 mg/kg), and cilostazol (20 mg/kg). (**A**) Testicular malondialdehyde, (**B**) total nitrite level, (**C**) reduced glutathione levels in rats treated with different doses of cilostazol, (**D**) testicular malondialdehyde, (**E**) total nitrite level, and (**F**) reduced glutathione levels of various phosphodieterase inhibitor groups. Data are represented as means ± S.E.M. of 6 rats per group. *, #, ●, †, !, @ are significant (*p* < 0.05) differences from control, CIS, CIS + Cilo 5 mg, CIS + Cilo 10 mg, CIS + TDF, and CIS + PTX groups, respectively. CIS: cisplatin; TDF: tadalafil; PTX: pentoxifylline; Cilo: cilostazol.

Lipid peroxides and total nitrite levels were significantly elevated in testicular homogenates of CIS rats, while the antioxidant capacity of GSH was significantly attenuated (Figure 2A–C).

However, testicular MDA and total nitrite levels significantly diminished with all examined drugs compared to CIS-intoxicated rats. Notably, the 20 mg/kg dose of Cilo suppressed testicular total nitrite contents even more than the 5 or 10 mg/kg Cilo treatments.

Consistent with previous results, treatment with Cilo significantly increased the content of GSH. In addition, a high dose of Cilo (20 mg/kg) produced a more enhanced response in GSH capacity compared to groups treated with lower doses (5 and 10 mg/kg).

Moreover, 20 mg of Cilo, compared to TDF and PTX, had a greater effect on lowering total nitrite contents. This effect went along with the GSH capacity values, which showed a significant increase in GSH in the CIS + Cilo 20 mg group compared to the CIS + TDF and CIS + PTX groups.

### 2.4. Effects on Testicular Inflammatory Mediators (TNF-α and NF-κB) Protein Expressions

**Figure 3:**
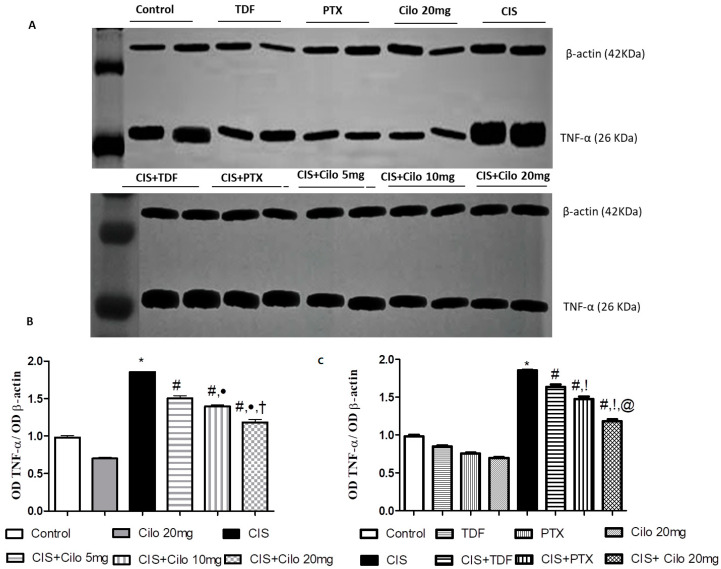
Representative Western blots showing the effect of cilostazol (5, 10 and 20 mg/kg), tadalafil (5 mg/kg), and pentoxifylline (75 mg/kg) on TNF-α protein expression in testicular tissues of CIS-treated rats. (**A**) Representative Western blots showing target protein bands from each group. (**B**) Quantified densitometric analysis of testicular TNF-α protein expression in CIS groups treated with cilostazol (5, 10, and 20 mg/kg). (**C**) Comparison between the levels of TNF-α in rat testes of CIS groups treated with tadalafil (5 mg/kg), pentoxifylline (75 mg/kg), and cilostazol (20 mg/kg). Values for each bar represent the means ± S.E.M., the ratio of densitometric measurements (OD) of samples to the corresponding β-actin. *, #, ●, †, !, @ are significant (*p* < 0.05) differences from the control, CIS, CIS + Cilo 5 mg, CIS + Cilo 10 mg, CIS + TDF, and CIS + PTX groups, respectively. TNF-α: tumor necrosis factor-alpha; CIS: cisplatin; TDF: tadalafil; PTX: pentoxifylline; Cilo: cilostazol.

**Figure 4:**
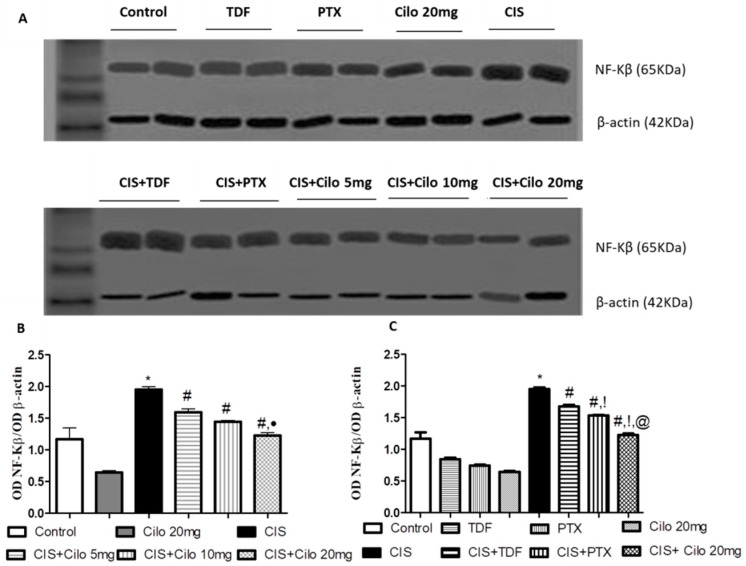
Representative Western blots showing the effect of cilostazol (5, 10, and 20 mg/kg), tadalafil (5 mg/kg), and pentoxifylline (75 mg/kg) on NF-κB protein expression in testicular tissues of CIS-treated rats. (**A**) Representative Western blots showing target protein bands from each group. (**B**) Quantified densitometric analysis of testicular NF-κB protein expression in CIS groups treated with cilostazol (5, 10, and 20 mg/kg). (**C**) Comparison between the levels of NF-κB in rat testes of groups treated with tadalafil (5 mg/kg), pentoxifylline (75 mg/kg), and cilostazol (20 mg/kg). Values for each bar represent the means ± S.E.M., the ratio of densitometric measurements (OD) of samples to the corresponding β-actin. *, #, ●, !, @ are significant (*p* < 0.05) differences from control, CIS, CIS + Cilo 5 mg, CIS + Cilo 10 mg, CIS + TDF, and CIS + PTX groups, respectively. NF-κB: nuclear factor-kappa B; CIS: cisplatin; TDF: tadalafil; PTX: pentoxifylline; Cilo: cilostazol.

Protein expressions of NF-κB and pro-inflammatory cytokines (TNF-α) in testicular tissues were examined with Western blot analysis. As shown in Figure 3 and Figure 4, TNF-α and NF-κβ protein expression in testes was significantly elevated in the CIS groups compared to control animals. However, different interventions significantly blunted CIS-induced abnormalities in the levels of these parameters. Moreover, the reduction in TNF-α and NF-κβ expression was more pronounced in CIS rats treated with 20 mg/kg of Cilo compared to either the CIS + Cilo 5 mg or the CIS + Cilo 10 mg group.

In addition, testicular TNF-α and NF-ĸB protein expression significantly decreased in the CIS + Cilo 20 mg group when compared with their levels in groups treated with other PDE inhibitors.

### 2.5. Effects on Testicular Caspase-3 Protein Expression

**Figure 5:**
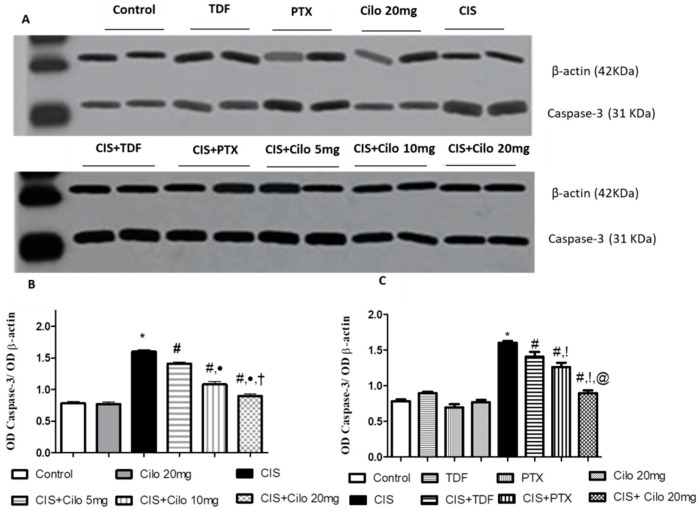
Representative Western blots showing the effect of cilostazol (5, 10, and 20 mg/kg), tadalafil (5 mg/kg), and pentoxifylline (75 mg/kg) on caspase-3 protein expression in testicular tissues of CIS-treated rats. (**A**) Representative Western blots showing target protein bands from each group. (**B**) Quantified densitometric analysis of testicular caspase-3 protein expression in CIS groups treated with cilostazol (5, 10, and 20 mg/kg). (**C**) Comparison between the levels of caspase-3 in rat testes of groups treated with tadalafil (5 mg/kg), pentoxifylline (75 mg/kg), and cilostazol (20 mg/kg). Values for each bar represent the means ± S.E.M., the ratio of densitometric measurements (OD) of samples to the corresponding β-actin. *, #, ●, †, !, @ are significant (*p* < 0.05) differences from control, CIS, CIS + Cilo 5 mg, CIS + Cilo 10 mg, CIS + TDF, and CIS + PTX groups, respectively. CIS: cisplatin; TDF: tadalafil, PTX: pentoxifylline; Cilo: cilostazol.

The protein expression level of caspase-3, a diagnostic apoptotic marker, significantly increased in the model group. This upregulation of caspase-3 expression was significantly counteracted in all groups treated with Cilo in a dose-dependent manner, as well as the other investigated PDE inhibitors, TDF and PTX (Figure 5).

On comparing the effect of 20 mg of Cilo with TDF and PTX on caspase-3 protein expression, it was found that 20 mg of Cilo significantly abolished the upregulation of caspase-3 expression caused by CIS relative to the CIS + TDF and CIS + PTX groups.

## 3. Discussion

The clinical application of CIS is greatly restricted due to its hazards, particularly testicular toxicity. This study demonstrated, for the first time, that the PDE-3 inhibitor Cilo promotes a potential protective effect against CIS-induced testicular damage, as evident by a marked increase in serum testosterone, sperm motility, and sperm count, as well as a reduction in the percentage of abnormal sperm. Abrogation of oxidative stress along with a reduction in NF-κB, TNF-α, and caspase-3 expression might contribute to Cilo’s therapeutic effect. Moreover, Cilo afforded a stronger effect on dampening male reproductive toxicity caused by CIS than that exerted by the other investigated PDE inhibitors, TDF and PTX.

Here, CIS-intoxicated rats exhibited a marked decline in the relative testicular weight, a frequently measured indicator of spermatogenesis [5]. Moreover, CIS administration led to a significant low epididymal sperm count, impaired sperm motility, and increased sperm morphological abnormalities with a concomitant decline in the testosterone level. The histopathological findings that showed desquamated and disorganized germinal cells as well as degeneration in germinal cells confirmed the deterioration of sperm quality indices in rats administered CIS alone. These observed effects of CIS were consistent with the results of previous studies [4,5,14,15,22]. However, pretreatment with TDF, PTX, and three doses of Cilo restored the loss of testicular weight and enhanced the number and motility of epididymal sperm, as well as serum testosterone levels, compared to the CIS group. Also, Cilo-treated groups showed marked improvement in the histopathological architecture in a dose-dependent manner. These results are consistent with previous studies that reported the ability of Cilo to mitigate testicular dysfunction caused by diabetes and testicular ischemia reperfusion injury [11,21].

The potential of TDF and PTX to ameliorate CIS-induced spermatogenic toxicity has been demonstrated in previous studies [13,15]. Of interest, serum testosterone levels, sperm indices, and histological alteration significantly improved in the CIS + Cilo 20 mg group compared to CIS-intoxicated rats treated with other PDE inhibitors. These observations provide insights into the possible effect of Cilo on abrogating testicular dysfunction induced by CIS. Based on these outcomes, we aimed to investigate molecular mechanisms that could partially illustrate the testicular preservative effect of Cilo in a CIS setting.

Despite the lack of knowledge of an exact mechanism explaining CIS-induced cellular damage in testicular tissues, mechanistic studies have revealed that oxidative stress remains the hallmark mechanism involved in CIS testicular toxicity [23]. It is clearly demonstrated that there is a strong relationship between normal spermatozoa function and balanced oxidative status [24,25]. In addition, CIS mitigates testosterone production via an ROS-inhibited P450 side-chain cleavage enzyme [26]. In accordance with several reports [14,22,27], impaired oxidative status in testicular tissues was presented in this work by a significant elevation in MDA content, a marker of lipid peroxidation, and total nitrite levels. In cases of increased oxidative stress, the polyunsaturated phospholipids of the cell membrane are oxidized, generating MDA. Hence, its elevated level indicates increased oxidative stress [23].

Moreover, increased ROS generation caused by CIS exceeded the antioxidant defense capacity, which subsequently reduced these antioxidant tools and presented a dramatic depletion in GSH levels.

More importantly, the administration of Cilo dampened oxidative stress, which was confirmed by significantly lower MDA and nitrite levels and a higher GSH concentration. This effect was achieved with three different doses of Cilo, but the higher dose afforded more pronounced antioxidant activity, which indirectly highlights the dose-dependent antioxidant effect of Cilo. These results can be explained in the light of published reports indicating that Cilo treatment significantly attenuated lipid peroxidation and restored GSH capacity in testes in a streptozotocin diabetic model [11] and testicular ischemia reperfusion [21]. Also, it was demonstrated that milrinone, another PDE3 inhibitor, significantly attenuated MDA [12].

The antioxidant effect of TDF and PTX was demonstrated here by a significant decrease in testicular MDA and total nitrite levels, as well as an increase in GSH capacity, which is consistent with previous reports [13,14,15,28,29]. The improvement in sperm motility produced by PTX in infertile patients is mediated by the mitigation of oxidative stress [30]. More importantly, the inhibitory effect of Cilo at high doses on oxidative stress was more profound relative to TDF or PTX therapy, which indirectly highlights that therapy with Cilo, a PDE inhibitor, may be an effective regimen for alleviating testicular injury by scavenging free radicals.

Additionally, exaggerated ROS generation evokes the inflammatory cascades that are clearly involved in the pathogenesis of CIS spermatoxicity and gonadotoxicity [31,32]. In this context, this study showed that exposure to CIS results in significant expression of inflammatory mediators, TNF-α and NF-κB proteins, in testicular tissue compared to the control group. Previous investigations have reported that rats treated with CIS show testicular inflammation, which was evident by the overexpression of inflammatory mediators, such as TNF-α, NF-κB, and inducible nitric oxide synthase (iNOS) [1,31,33,34].

It is worth mentioning that elevated total nitrite levels could be attributed to TNF-α’s capability to upregulate iNOS expression, which subsequently increases NO generation [35]. Elevated NO levels, in turn, react with superoxide anion to produce peroxynitrite radicals that trigger harmful effects, causing testicular toxicity [36].

In contrast, treatment with Cilo (5, 10, or 20 mg/kg) could ameliorate upregulated TNF-α and NF-κB expressions induced by CIS. This result is in accordance with earlier studies that have reported the anti-inflammatory properties of Cilo in different experimental models of testicular injury [11,21]. Furthermore, the protective effect of Cilo against diclofenac-induced renal toxicity [37], high-fat-diet-induced nonalcoholic fatty liver [38], and cyclophosphamide-induced cardiac damage [39] has been partially attributed to its ability to diminish the level of inflammatory markers. Milrinone also dramatically decreases TNF-α levels, which contributes to its ameliorative effect against ischemia-reperfusion testicular injury [12].

Moreover, the inhibitory effect of Cilo on Toll-like receptor (TLR) signaling-mediated NF-κB activation could explain its anti-inflammatory effects. Cilo also causes a direct interruption of NF-κB recruitment to promoters of pro-inflammatory genes, which subsequently reduces TLR-mediated transcriptional activation of pro-inflammatory genes [40,41]. The observed anti-inflammatory properties of Cilo may also be attributed to increased cellular levels of cAMP, which in turn suppresses the production of TNF-α [42]. Notably, inflammation was further halted with a higher dose of Cilo (20 mg/kg).

Of interest, inhibition of PDE with TDA and PTX exhibited anti-inflammatory properties in CIS-induced male reproductive toxicity, which was evident by the significant downregulation of testicular TNF-α and NF-κB protein expression. These findings were consistent with previous studies. The anti-inflammatory effect of TDF partially explains its ability to alleviate CIS-induced reproductive toxicity [13] and thioacetamide-induced liver fibrosis in rats [43]. Further, the potent anti-inflammatory properties of PTX have been documented in various illnesses [44]. PTX decreased TNF-α and NF-κB in cerebral ischemia reperfusion [45], high-fat-diet-induced nonalcoholic fatty liver [38], and diclofenac-induced acute renal injury [37] models. Importantly, the decline in NF-κB and TNF-α expression was more pronounced in CIS rats treated with 20 mg of Cilo compared to either the TDF or the PTX group.

Oxidative stress, with its cascades of increasing expression of inflammatory mediators, has been shown to induce cell death in the testes of animals exposed to CIS either via necrosis or via apoptosis [31,46]. In vivo and in vitro studies have demonstrated that the imbalance of mitochondrial redox processes caused by CIS leads to cell death associated with caspases [47,48]. Excess ROS mediates Ca^2+^ influx, which subsequently activates apoptotic processes [49]. Translocation of cytosolic Bcl-2-associated X protein (Bax), a pro-apoptotic factor, to the mitochondria and imbalance of the Bax/B-cell lymphoma 2 (Bcl-2), an anti-apoptotic factor, ratio induced by CIS result in caspase-3 activation. These events together contribute to cell apoptosis upon exposure to CIS [50].

In this study, significant elevations in apoptosis-related proteins, including caspase 3, were displayed in the testes of the CIS group. In contrast, different PDE inhibitors protected the rats against CIS-induced testicular apoptosis, as supported by a significant decline in pro-apoptotic marker protein expression when compared to CIS alone. The anti-apoptotic activity of Cilo has been demonstrated in several experimental models, including cyclophosphamide-induced ovarian toxicity [51], testicular ischemia/reperfusion [21], and cochleo-vestibular dysfunction induced by CIS [52]. This property of Cilo could be attributed to its activating cAMP-dependent protein kinase. The anti-apoptotic impact of TDF and PTX has also been observed in previous studies [13,29,53,54]. Interestingly, the higher dose of Cilo (20 mg/kg) caused further attenuation of testicular caspase-3 expression compared to lower doses (5 and 10 mg/kg) of Cilo and in groups treated with TDF and PTX.

To that end, we collectively propose that treatment with the PDE3 inhibitor Cilo may provide marked protection over the use of TDF or PTX against CIS-induced testicular injury. The protective effect of Cilo can possibly be attributed to the restoration of an unbalanced oxidative status alongside anti-inflammatory and anti-apoptotic effects.

## 4. Materials and Methods

### 4.1. Drugs and Chemicals

Cisplatin, TDF, PTX, and Cilo were purchased from Bristol Mayers Co. (Princeton, NJ, USA), Eva Pharma for Pharmaceutical Industries (Cairo, Egypt), Medical Union Pharmaceutical (Cairo, Egypt), and Pharmacare for Pharmaceutical Industries (Egypt), respectively. Polyclonal rabbit/anti-rat primary antibody against TNF-α was obtained from Thermo Fischer Scientific Inc./Lab Vision (Fermont, CA, USA). Antibodies against nuclear factor-kappa B (NF-κB) and caspase-3 were purchased from Biolegend (San Diego, CA, USA) and NSJ Bioreagents (San Diego, CA, USA), respectively. All other chemicals of analytical grade were obtained from Sigma-Aldrich (Dorset, Germany).

### 4.2. Experimental Design

Seventy male Wistar rats, weighing 180–210 g, obtained from the Animal Care Unit, Faculty of Pharmacy, Nahda University, were used in this study. Throughout the experiment period, they were housed in a constant environment of a 12 h/12 h dark/light cycle and a temperature of 25 °C ± 2 °C. The animals were allowed to feed on commercially available chow pellet diets and water ad libitum.

Before the beginning of the experiment, the animals were allowed to adapt in the lab for 1 week. After that, 28 rats were randomly selected as control groups (7 rats each) and received the corresponding vehicle: the TDF group was administered TDF (5 mg/kg/day, P.O.), the PTX group was administered PTX (75 mg/kg/day, P.O.), and the Cilo 20 mg group received Cilo (20 mg/kg/day, P.O.) for 14 successive days. The remaining (n = 42) rats were randomly divided into 6 groups (7 rats each) and treated for the duration of 14 consecutive days as follows:

The CIS group was treated with a single i.p. injection of CIS (7 mg/kg) dissolved in saline, a dose that is sufficient to induce testicular injury in male rats according to previous studies [1,5,55], on the 7th day of the experiment.

The CIS + TDF group was given TDF (5 mg/kg/day, P.O.) for 14 successive days, while being injected with CIS (7 mg/kg, i.p.) on the 7th day of the experiment.

The CIS + PTX group received PTX (75 mg/kg/day, P.O.) for 14 successive days, while being injected with CIS (7 mg/kg, i.p.) on the 7th day of the experiment.

The CIS + Cilo 5 mg group received Cilo (5 mg/kg/day, P.O.) for 14 successive days, while being injected with CIS (7 mg/kg, i.p.) on the 7th day of the experiment.

The CIS + Cilo 10 mg group was treated with Cilo (10 mg/kg/day, P.O.) for 14 successive days, while being injected with CIS (7 mg/kg, i.p.) on the 7th day of the experiment.

The CIS + Cilo 20 mg group was exposed to the same regimen as the previous two groups except for a higher dose of Cilo (20 mg/kg/day, P.O.).

TDF and PTX were dissolved in normal saline, while Cilo was dissolved in a carboxy methyl cellulose solution. The dosages and timings for TDF and PTX administration were based on previous reports [13,15]. Meanwhile, doses of Cilo were selected based on previous pharmacological studies. Cilo at these doses has been reported to suppress reproductive toxicity induced by cyclophosphamide, testicular ischemia/reperfusion, or streptozotocin [11,21,51].

### 4.3. Collection of Samples

At the end of the experiment, 12-h-fasted rats were anesthetized with pentobarbital sodium (50 mg/kg, i.p.). Collection of blood samples was performed via cardiac puncture, and to separate the sera, blood samples were centrifuged for 10 min at 3000 rpm to measure testosterone levels. Testes were excised, rinsed, and dried. The testes were weighed to calculate the relative testicular weight (testicular weight/body weight × 100). One testis of each animal was fixed in 10% formalin for histological examination. The other one was flash-frozen in liquid nitrogen, stored at −80 °C, and subsequently homogenized in cold potassium phosphate buffer (0.05 M, pH 7.4) for various biochemical and Western blot analyses.

### 4.4. Sperm Motility and Count

Assessment of the epididymal sperm motility and count was performed immediately. With the aid of TOX IVOS II, the total sperm number was determined by Kenjale et al. [56]. The dissected cauda epididymis was weighed, minced in 5 mL of physiological saline immediately, and then incubated for 30 min at 37 °C, allowing sperm to leave the tubules of the epididymis. A phase contrast microscope at 400× magnification was used to record the percentage of motile sperm from the cauda epididymis left. The total number of sperm was then calculated.

### 4.5. Determination of the Serum Testosterone Level

The testosterone level in the serum was measured using a commercial rat-specific ELISA kit following the manufacturer’s guidelines (catalog # CSB-E05100r; Cusabio Biotech Co., Ltd., Wuhan, China).

### 4.6. Determination of Testicular Oxidative Stress Markers

The antioxidant status in the testes was screened through the determination of malondialdehyde (MDA), a lipid peroxidation product; nitric oxide (NO); and reduced glutathione (GSH) in the testicular homogenate. The process of MDA measurement is based on the reaction of MDA with thiobarbituric acid to form a pink-colored complex assessed spectrophotometrically at 535 nm [57]. The testicular NO was quantified as total nitrate/nitrite (the stable degradation products of NO). By using copperized cadmium, nitrate is reduced to nitrite, followed by color development in an acidic medium with Griess reagent [58]. The determination of the GSH concentration was performed using a commercially available spectrophotometric kit (Biodiagnostic, Cairo, Egypt). The thiol component of GSH reduces Ellman’s reagent (5,5-dithio-bis-2-nitrobenzoic acid), resulting in a yellow-colored compound (5-thio-2-nitrobenzoic acid), which was measured spectrophotometrically at 405 nm.

### 4.7. Western Blot Analysis

Frozen testicular tissue samples were homogenized in ice-cold lysis buffer (Bio BASIC INC., Markham, ON, Canada). After quantitation of protein concentrations using the established Bradford dye-binding method (Bio-Rad, Hercules, CA, USA), aliquots of the lysate with equal protein amounts were electrophoresed on sodium dodecyl sulfate–polyacrylamide gel electrophoresis (SDS-PAGE).

Following protein extraction, proteins were transferred to polyvinyl difluoride (PVDF) membranes. After incubation of the membranes in a blocking solution containing 5% (*w*/*v*) non-fat milk, they were incubated with primary antibodies against the blotted target protein, TNF-α (catalog # PA5-19810, dilution 1:3000), NF-κB (catalog # 622601, dilution 0.05 μg/mL), and caspase-3 (catalog # R31602, dilution 0.5 mg/mL) diluted in blocking buffer at 4 °C overnight. After extensive rinsing, the membranes were incubated with a secondary antibody (goat anti-rabbit IgG; Novus Biologicals, Wiesbaden-Nordenstadt, Germany) conjugated to horseradish peroxidase for 1 h. Protein bands were visualized with a standard enhanced chemiluminescent method. The densitometry measurements of the target protein band relative to those of the corresponding β-actin band were semi-quantified, presented as a ratio of the relative optical density, and calibrated as fold-change values from the control using Image J 1.54d software (freeware; rsbweb.nih.gov/ij).

### 4.8. Histopathological Examination

The testes collected from all experimental animals were washed with normal saline and fixed in Bouin’s solution for 1–2 days. After that, the testes were dehydrated in a graded ethanol series, then cleared in xylene, and embedded in paraffin blocks. The blocks were sectioned at 4–6 µm thickness. The obtained sections were deparaffinized with xylol and stained with hematoxylin and eosin (H&E) for routine histopathological examination under a light microscope, according to Suvarna [59]. In addition, the microscopic scoring for qualitative histopathological changes in seminiferous tubules was graded on a scale of (−) absence of pathological finding, (+) mild, (++) moderate, and (+++) severe. Mild, moderate, and severe represented pathological findings of <25%, 25–50%, and >50% of the total examined area of seminiferous tubules, respectively. Scoring was applied on the following histological parameters: desquamation and disorganization in germinal cells, interstitial edema, degeneration in germinal cells, and reduction in germinal cell counts [60].

### 4.9. Statistical Analysis

Data were expressed as means ± S.E.M. and statistically analyzed through one-way analysis of variance (ANOVA); next, comparisons between individual groups were performed using the Tukey–Kramer post-analysis test. The results were considered statistically significant when differences were significant at a probability (*p*) value of <0.05. Statistical analysis was performed using Graph Pad Prism^®^ software (version 5.0 for Windows; Graph Pad Software, San Diego, CA, USA).

## 5. Conclusions

The findings of this study suggest that Cilo has a dose-dependent positive impact on male reproductive dysfunction in CIS-intoxicated rats. The protective effect of Cilo is possibly explained through free-radical scavenging. We also shed light on the significance of molecular mechanisms involved in the protective role of this drug by targeting the inflammatory and cell death signaling pathways through the reduction in TNF-α, NF-κB, and caspase-3 expressions. This invites clinical studies to evaluate the protective role of Cilo in cancer patients treated with CIS to prevent the incidence of reproductive toxicity.

## Figures and Tables

**Table 1:** Effect of cilostazol (5, 10, and 20 mg/kg), tadalafil (5 mg/kg), and pentoxifylline (75 mg/kg) on CIS-induced changes in relative testicular weight, testosterone concentration, and sperm quality indices.

Groups (dose: mg/kg)	Relative Testicular Weight	Serum Testosterone Level (ng/mL)	Sperm Count (×10^6^/mm^3^)	Sperm Motility (%)	Abnormal Sperm Morphology (%)
Control	0.69 ± 0.044	2.49 ± 0.059	38.50 ± 0.71	72.67 ± 1.53	12.67 ± 1.52
TDF 5	0.63 ± 0.027	2.58 ± 0.031	38.00 ± 1.00	68.00 ± 1.41	14.00 ± 1.00
PTX 75	0.65 ± 0.033	2.63 ± 0.042	35.00 ± 2.65	68.50 ± 0.71	15.00 ± 2.00
Cilo 20	0.65 ± 0.069	2.74 ± 0.042	35.33 ± 1.15	68.00 ± 1.40	15.50 ± 0.71
CIS	0.49 ± 0.028 *	0.93 ± 0.051 *	13.66 ± 1.53 *	32.67 ± 1.52 *	35.00 ± 1.41 *
CIS + TDF	0.64 ± 0.027 ^#^	1.15 ± 0.059 ^#^	20.66 ± 0.58 ^#^	38.00 ± 1.00 ^#^	27.67 ± 0.57 ^#^
CIS + PTX	0.73 ± 0.064 ^#^	1.46 ± 0.044 ^#,!^	23.00 ± 1.00 ^#^	41.00 ± 1.00 ^#^	24.67 ± 1.53 ^#^
CIS + Cilo 5	0.73 ± 0.027 ^#^	1.71 ± 0.011 ^#^	25.50 ± 0.71 ^#^	42.67 ± 1.51 ^#^	23.50 ± 0.71 ^#^
CIS + Cilo 10	0.75 ± 0.061 ^#^	1.84 ± 0.040 ^#,●^	26.66 ± 1.52 ^#^	49.00 ± 2.00 ^#,●^	19.00 ± 1.00 ^#,●^
CIS + Cilo 20	0.70 ± 0.098 ^#^	1.97 ± 0.020 ^#,●,†,!,@^	31.50 ± 0.70 ^#,●,†,!,@^	59.67 ± 1.52 ^#,●,†,!,@^	19.00 ± 0.99 ^#,●,!,@^

Data are represented as means ± S.E.M. of 6 rats per group. *, #, ●, †, !, @ are significant (*p* < 0.05) differences from control, CIS, CIS + Cilo 5 mg, CIS + Cilo 10 mg, CIS + TDF, and CIS + PTX groups, respectively. CIS: cisplatin, TDF: tadalafil, PTX: pentoxifylline, Cilo: cilostazol.

**Table 2:** Histopathological scoring of testicular lesions summarizing the effect of cilostazol (5, 10, and 20 mg/kg), tadalafil (5 mg/kg), and pentoxifylline (75 mg/kg) on histopathological changes in CIS-induced testicular injury.

Lesion Groups (dose: mg/kg)	Desquamation in Germinal Cells	Disorganization in Germinal Cells	Interstitial Edema	Degeneration in Germinal Cells	Reduction in Germinal Cell Counts
Control	-	-	-	-	-
TDF 5	-	-	-	-	-
PTX 75	-	-	-	-	-
Cilo 20	-	-	-	-	-
CIS 7	++	++	+	++	++
CIS + TDF	++	+	+	++	+
CIS + PTX	+	+	++	+	+
CIS + Cilo 5	++	++	+	+	+
CIS + Cilo 10	++	++	+	+	+
CIS + Cilo 20	-	-	+	+	-

Score level of (-) means the absence of histological changes. Scores of mild (+) and moderate (++) refer to the appearance of histopathological alterations in 25% or less than 50% of the total examined fields, respectively. CIS: cisplatin; TDF: tadalafil; PTX: pentoxifylline; Cilo: cilostazol.

## Data Availability

All data and materials are available from the corresponding author upon request.
